# Chondrosarcoma of the Pelvis: Oncologic and Functional Outcome

**DOI:** 10.1080/13577140020025878

**Published:** 2000-12

**Authors:** Reiner J. Wirbel, Michael Schulte, Bernd Maier, Martin Koschnik, Wolf E. Mutschler

**Affiliations:** 1 Department of Trauma Hand and Reconstructive Surgery University of Saarland Homburg Germany; 2 Department of Trauma Hand, Plastic and Reconstructive Surgery University of Ulm Ulm Germany; 3 Klinik für Unfallchirurgie St.-Elisabeth-Krankenhaus Friedrich Ebert Strase 59 Neuwied D-56564 Germany

## Abstract

*Purpose.* Chondrosarcoma (CS) most commonly involves the pelvis. The factors that influence local and systemic control of pelvic CS and the functional outcome should be evaluated.

*Patients.* Fifty-one patients (37 males and 14 females; mean age, 39.4 years) with pelvic CS were included in this retrospective study.

*Methods.* The tumor stage, surgical treatment, surgical margin achieved, complications, incidence of local recurrence (LR), incidence of distant metastases, and the oncologic and functional status were evaluated. Oncologic outcome was estimated by the method of Kaplan and Meier, and the functional status was scored according to Musculoskeleral Tumor Society (MSTS) criteria. Analysis of variance was used to determine the factors that influence the oncologic and functional outcome.

*Results.* Surgical stages were IA in three cases, IB in 23, IIB in 23, and III in two. Hemipelvectomy (H) was performed in 13 cases, internal hemipelvectomy (IH) with endoprosthetic replacement in 17, and continuity resection (CR) in 23.Two patients received IH and CR, one due to LR, and one due to instability. Radical or wide margins were achieved in 27 cases, marginal margins in 16, and intralesional margins in eight. Local complication required additional surgery in 10 cases due to local infections and/or hematomas.Two patients died perioperatively. In 48 out of the 49 remaining patients, follow-up was available with a mean duration of 73.4 months (range, 4–229 months).Twenty patients died of the disease, two patients are alive with metastases, four patients are disease free after LR, and 22 patients show no evidence of the disease. LR occurred in 10 cases (20.4%), and 17 patients (34.6%) developed distant metastases. Functional evaluation of the 28 survivors revealed good and excellent results in 19 cases, fair in three and poor in six.The mean MSTS score of all survivors was 69.2%, after H it was 37.6%, after IH was 61.4%, and after CR was 79.5%.

*Conclusion.* In pelvic chondrosarcoma, survival was determined by the tumor stage and the surgical margin achieved.The incidence of LR was influenced by the surgical margin achieved, whereas the incidence of distant metastases was influenced by the tumor stage. The best oncologic results in chondrosarcoma involving the innominate bone could be found in low-grade tumors, and the best functional results after continuity resection and restoration of the pelvic girdle.


*Sarcoma* (2000) **4**, 161–168

ORIGINAL ARTICLE

### Chondrosarcoma of the pelvis: oncologic and functional outcome

REINER J. WIRBEL,[1] MICHAEL SCHULTE,[2] BERND MAIER,[1] MARTIN KOSCHNIK[1] & WOLF E. MUTSCHLER[1]

[1]*Department of Trauma, Hand and Reconstructive Surgery, University of Saarland, Homburg, Germany,* [2]*Department of Trauma, Hand, Plastic and Reconstructive Surgery, University of Ulm, Ulm, Germany*

**Abstract**

*Purpose.* Chondrosarcoma (CS) most commonly involves the pelvis. The factors that influence local and systemic control of pelvic CS and the functional outcome should be evaluated.

*Patients.* Fifty-one patients (37 males and 14 females; mean age, 39.4 years) with pelvic CS were included in this retrospective study.

*Methods.* The tumor stage, surgical treatment, surgical margin achieved, complications, incidence of local recurrence (LR), incidence of distant metastases, and the oncologic and functional status were evaluated. Oncologic outcome was estimated by the method of Kaplan and Meier, and the functional status was scored according to Musculoskeleral Tumor Society (MSTS) criteria. Analysis of variance was used to determine the factors that influence the oncologic and functional outcome.

*Results.* Surgical stages were IA in three cases, IB in 23, IIB in 23, and III in two. Hemipelvectomy (H) was performed in 13 cases, internal hemipelvectomy (IH) with endoprosthetic replacement in 17, and continuity resection (CR) in 23. Two patients received IH and CR, one due to LR, and one due to instability. Radical or wide margins were achieved in 27 cases, marginal margins in 16, and intralesional margins in eight. Local complication required additional surgery in 10 cases due to local infections and/or hematomas. Two patients died perioperatively. In 48 out of the 49 remaining patients, follow-up was available with a mean duration of 73.4 months (range, 4–229 months). Twenty patients died of the disease, two patients are alive with metastases, four patients are disease free after LR, and 22 patients show no evidence of the disease. LR occurred in 10 cases (20.4%), and 17 patients (34.6%) developed distant metastases. Functional evaluation of the 28 survivors revealed good and excellent results in 19 cases, fair in three and poor in six. The mean MSTS score of all survivors was 69.2%, after H it was 37.6%, after IH was 61.4%, and after CR was 79.5%.

*Conclusion.* In pelvic chondrosarcoma, survival was determined by the tumor stage and the surgical margin achieved. The incidence of LR was influenced by the surgical margin achieved, whereas the incidence of distant metastases was influenced by the tumor stage. The best oncologic results in chondrosarcoma involving the innominate bone could be found in low-grade tumors, and the best functional results after continuity resection and restoration of the pelvic girdle.

**Key words:** *chondrosarcoma, pelvis, functional outcome*

### Introduction

Chondrosarcoma is a malignant tumor, consisting of cartilaginous tissue without osteoid, and accounts for approximately 20% of bone sarcomas.[1–3] The tumor arises predominantly in the middle-aged population and occurs most commonly in the pelvis and the upper end of the femur.[1–7]

Primary bone sarcomas of the pelvis are considered to have a worse prognosis compared with those located in the long bones.[8–14] There exist several reports of chondrosarcoma involving the innominate bone, and most of them have been focused primarily on reconstruction surgery.[4–7,14–16]

However, the prognosis of this common pelvic tumor is described by only a few authors.[6,7,9–11,15]

Since chemotherapy and irradiation are not effective, surgery achieving an adequate, i.e. wide, margin is currently the only known effective treatment of chondrosarcoma.

However, in pelvic lesions, and adequate margin is often not feasible because of the extent of the tumor and the lack of compartimental barriers. Frequent local and/or systemic failures after surgical treatment complicate the outcome in these patients.

The purpose of this study was to define factors that influence local and systemic control of pelvic chondrosarcoma and to evaluate the functional results.

Correspondence to: Dr R. J. Wirbel, Klinik für Unfallchirurgie, St.-Elisabeth-Krankenhaus, Friedrich Ebert Strase 59, D-56564 Neuwied, Germany. Tel: +49 2631 821389; Fax: +49 2631 821624.

1357-714X print/1369-1643 online/00/040161-08 © 2000 Taylor & Francis Ltd
*DOI: 10.1080/13577140020025878*

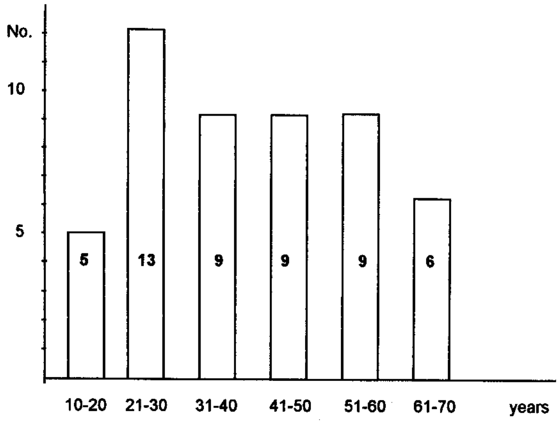

**Fig. 1.** *Age distribution of 51 patients with pelvic chondrosarcoma.*

### Patients

### *Patients*

Fifty-one patients with chondrosarcoma involving the innominate bone were treated between October 1978 and April 1998. There were 37 males and 14 females with an average age of 39.4 years (range, 12–64 years). The age distribution is shown in Figure 1.

The anatomical origin of the tumor was located in the illium in 26 cases, in the acetabular region in 21, and in the pubis or ischium in four cases. According to the surgical staging system of the Musculoskeletal Tumor Society (MSTS),[17] there were three stage IA, 23 stage IB, 23 stage IIB, and two stage III lesions (Table 1).

Seven patients, three in stage IB, three stage IIB, and one in stage III, had previous surgical treatment in other institutions and presented with local recurrence. the median time to local recurrence in these cases was 48 months (range, 5–180 months).

The tumor volume was calculated according to the method of Göbel *et al.*[18] Tumors with a volume ≥ 500 ml were defined as large tumors, and < 500 ml were defined as small tumors. There were 21 small and 30 large tumors. The average tumor volume was 510 ml (range, 50–950 ml).

### *Treatment*

Fifty-three surgical procedures were performed and divided into three groups: 23 patients have undergone continuity resection (CR) of the tumor with or without restoration of the pelvic girdle (Fig. 2). The different types of CR were classified according to Enneking and Dunham.[5] Internal hemipelvectomy (IH) and endoprosthetic replacement was performed in 17 cases, when the tumor involved the acetabulum and the ilium or the pubis (Fig. 3). Hemipelvectomy (H) was necessary in 13 cases when the tumor invaded the iliac vessels or the sciatic nerve and, when sacrifiying a large amount of muscle, leaves the leg with poor function.

Two patients received, primarily, a CR and, secondarily, IH; in one case due to a local recurrence and in one due to instability after CR. Table 2 summarizes the different types of resections and reconstructions.

### Methods

Fifty patients could be followed-up routinely from the time of surgery to the time either of their death or the date of their most recent follow-up. In one patient, follow-up was not available. The screening for local recurrence and distant metastases includes X-ray of the chest and of the pelvis, and computed tomography (CT) scan of the pelvis. Patients with high-grade tumors (stage II) were controlled every 3 months for 2 years, and after this time twice a year for a further 3 years. In low-grade tumors, the screening was performed twice a year over a period of 5 years.

The mean follow-up was 73.4 months (range, 4–229 months). Data for this report was obtained from hospital records, maintained oncologic files and the clinical examination of the most recent follow-up. These include the different types of resections and reconstructions, the surgical margins achieved,[17] the tumor volume, the complications and need for re-operation, incidence of local recurrence and distant metastases, and the actual oncologic and functional status.

Excluding the two patients with stage III tumors, the Fisher exact test was used to compare the surgical margin obtained and the surgical treatment group (i.e. CR, IH, H), the surgical margins obtained and the incidence of local recurrence and metastases, the tumor volume and the surgical margin obtained, as well as the incidence of local recurrence and distant metastases, and the need for re-operation and the

**Table 1.** *Stage and origin of 51 patients with pelvic chondrosarcoma*

| | | Origin of the tumor | | |
|---|---|---|---|---|
| Stage | | Ileum | Acetabulum | Pubis or ischium |
| IA | 3 | | 3 | |
| IB | 23 | 15 | 7 | 1 |
| IIB | 23 | 10 | 10 | 3 |
| III | 2 | 1 | 1 | |
| Σ | 51 | 26 | 21 | 4 |

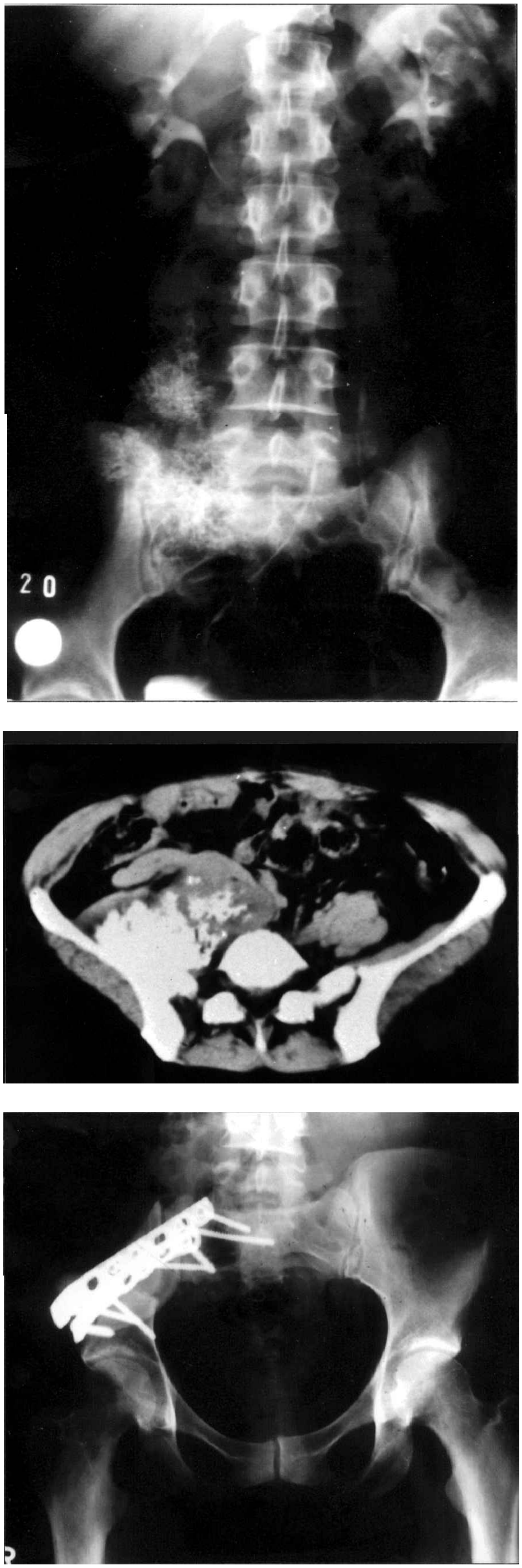

**Fig. 2.** *A 32-year-old woman with low-grade chondrosarcoma arising from the right ilium; (a) pre-operative X-ray showing calcifications and displacement of the right ureter, (b) computed tomography scan demonstrating the large extension of the tumor, and (c) postoperative X-ray after continuity resection (type IB) with restoration of the pelvic girdle by autograft and plates.*

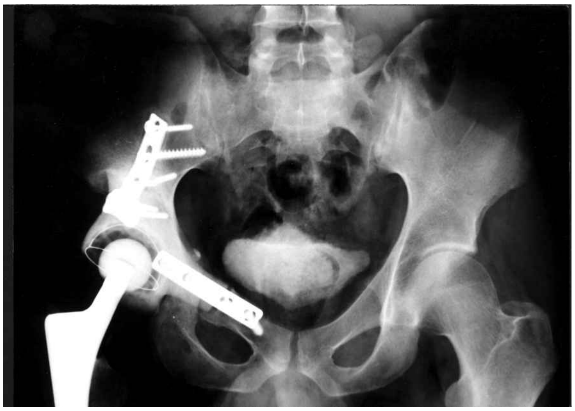

**Fig. 3.** *A 29-year-old man with low-grade chondrosarcoma arising from the right acetabulum; postoperative X-ray after internal hemipelvectomy and restoration of the pelvic girdle by polyacetal pelvic replacement.*

surgical treatment group. A $p$ value less than 0.05 was considered significant.

Using analysis of variance, the oncologic outcome was compared with the tumor stage, and the tumor volume, the origin of the tumor, the performed surgical procedure, and the surgical margin obtained. Overall survival was estimated by the method of Kaplan and Meier.[19]

The functional outcome obtained at the last clinical follow-up of the survivors was rated according to the most recent system of the MSTS.[20] This system includes the six following categories: pain, function of the hip joint, emotional acceptance, supports, walking ability, and gait analysis. A maximum of five points for each factor produces the maximum scores of 30 points. The patient's total score is divided by 30, resulting in a functional evaluation rate. The rating was considered excellent if five of the six factors scored five points, independent of the score of the sixth factor. A good rating was reported if five factors scored three points or more, and the sixth factor two points or less. A fair rating was regarded if five of the six factors scored one point or more, and the sixth factor zero points. If two or more factors scored zero points, the rating was considered poor.

### Results

### *Surgical procedure*

The 53 surgical procedures are listed in Table 2. All CR of type IB, IC and II required restoration of the pelvic girdle using auto- or allografts, plates, and/or composite fixations (Fig. 2). No reconstruction was necessary in type IA resection and in three out of five type III resections.

In 15 out of 17 patients, IH reconstruction was performed by polyacetal pelvic replacement[16] (Fig. 3), in one case a saddle prosthesis and in one case a computer-aided-designed pelvic replacement was used.

In three out of the 13 cases of H, extended

**Table 2**. *Surgical procedures*

| *n* (=53) | Resection | Reconstruction Total | None | Auto-/ allograft | Composite fixation | Plate |
|---|---|---|---|---|---|---|
| 23 | Continuity resection [5] | 3 type IA | 3 | | | |
| | | 6 type IB | | 4 | 1 | 1 |
| | | 6 type IX | | 1 | 4 | 1 |
| | | 1 type II | | | 1 | |
| | | 5 type III | 3 | 2 | | |
| 17 | Internal hemipelvectomy* | 15 polyacetal PR | | | | |
| | | 1 CAD-PR | | | | |
| | | 1 saddle prosthetic | | | | |
| 13 | Hemipelvectomy | | | | | |

*Two patients received internal hemipelvectomy after continuity resection. PR Pelvic replacement; CAD, computer-aided design.

procedures have to be performed: hemisacrectomy in two cases, in one of them with additional partial resection of the urinary bladder, and resection of the rectum with subsequent colostomy in one. Secondary hemipelvectomy was necessary in three cases; one due to local recurrence after CR, one due to local recurrence after IH, and one due to chronic infection after IH.

### *Surgical margin and tumor volume*

The surgical margins in relation to the tumor stage and the tumor volume are listed in Table 3. Margins were wide in 27 cases, marginal in 16, and intralesional in eight.

In 21 small tumors, 14 margins were wide, five marginal, and two intralesional. In 30 large tumors, 13 margins were wide, 11 marginal, and six intralesional. In 26 low-grade tumors, there were 19 wide and seven marginal margins. In 25 high-grade tumors, the margins were wide in eight cases, marginal in nine, and intalesional in eight.

There was no statistical difference between the tumor stage or volume and the obtainable surgical margin.

The margins that could be obtained in the different types of surgical procedure are demonstrated in Table 4. There was also no significant different between the obtainable surgical margin and the surgical treatment group.

### *Oncologic outcome*

The actual oncologic status in relation to the tumor stage, the surgical margin achieved, and the surgical treatment group is also listed in Table 4.

Twenty-two patients show no evidence of disease, four patients are disease free after local recurrence, two patients are alive with metastases, and 22 patients had died of disease. In one patient, follow-up and actual oncologic status was not known.

### *Local recurrence and metastases*

Excluding the two stage III and three stage IA tumors, Table 5 summarizes the incidence of local recurrence and distant metastases in relation to the tumor stage, surgical margin achieved, and the tumor volume.

Because two patients died perioperatively and follow-up was not available in one patient, the incidence of local recurrence and of metastases was calculated on the basis of 48 patients.

Local recurrence occurred in 10 cases (20.8%), with an average interval of 18.6 months (range 3–36

**Table 3**. *Stage, tumor volume and achieved surgical margin*

| Stage | *n* | Volume s (< 500 ml) | Volume l (≥ 500 ml) | Surgical margin w | Surgical margin m | Surgical margin i |
|---|---|---|---|---|---|---|
| IA | 3 | 3 | | 3 | | |
| IB | 23 | 11 | | 8 | 3 | |
| | | | 12 | 8 | 4 | |
| IIB | 23 | 7 | | 3 | 2 | 2 |
| | | | 16 | 5 | 7 | 4 |
| III | 2 | | 2 | | | 2 |
| Total | 51 | 18 | 33 | 27 | 16 | 8 |

s, small; l, large; w, wide; m, marginal; i, intralesional.

**Table 4**. *Oncologic outcome*

| | | Surgical procedure | | | Margin | | | Outcome | | | |
|---|---|---|---|---|---|---|---|---|---|---|---|
| Stage | *n* | CR | IH | H | w | m | i | NED | DFR | ADM | DOD |
| IA | 3 | 1 | 2 | | 3 | | | 3 | | | |
| IB | 23 | 15 | | | 10 | | | 10 | | | |
| | | | | | | 5* | | 1 | 2 | | 1 |
| | | | 6 | | 4 | | | 3 | 1 | | |
| | | | | | | 2 | | 1 | | | 1 |
| | | | | 2 | 2 | | | 2 | | | |
| IIB | 23 | 7 | | | 1 | | | | | | 1 |
| | | | | | | 3 | | | 1 | 1 | 1 |
| | | | | | | | 3 | | | | 3 |
| | | | 7 | | 4 | | | 1 | | | 3 |
| | | | | | | 3 | | 1 | | | 2 |
| | | | | 3 | 3 | | | | | 1 | 2 |
| | | | | | | 3 | 3 | | | | 6 |
| III | 2 | | | 2 | | | 2 | | | | 2 |
| Total | 51 | 23 | 15 | 13 | 27 | 16 | 8 | 22 | 4 | 2 | 22 |

CR, Continuity resection; IH, internal hemipelvectomy; H, hemipelvectomy; w, wide; m, marginal; i, intralesional; NED, no evidence of disease; DFR, disease free after local recurrence; ADM, alive with metastases; DOD, died of disease.
*In one of these patients, follow-up was not available.

**Table 5**. *Occurrence of local recurrence (LR) and metastases (Met) in relation to tumor stage and surgical margin achieved (stage III and IA tumors were excluded)*

| | | | | | Outcome | | |
|---|---|---|---|---|---|---|---|
| | *n* | LR | Met | LR+Met | DFR | ADM | DOD |
| Stage | | | | | | | |
| IB | 23 | 4 | | | 3 | | 1 |
| | | | 1 | | | | 1 |
| IIB | 23 | 3 | | | 1 | | 2 |
| | | 13 | | | 1 | 12 | |
| | | | | 3 | | 1 | 2 |
| Margin | | | | | | | |
| Wide | 24 | 1 | | | 1 | | |
| | | | 6 | | | 1 | 5 |
| | | | | 1 | | | 1 |
| Margin | 16 | 5 | | | 3 | | 2 |
| | | | 5 | | | | 5 |
| | | | | 1 | | 1 | |
| Intralesional | 6 | 1 | 3 | 1 | | | 5 |
| Volume | | | | | | | |
| Small | 15 | 2 | 4 | 1 | | | 7 |
| Large | 31 | 5 | | | 4 | | 1 |
| | | | 10 | | | 1 | 9 |
| | | | | 2 | | 1 | 1 |
| Total | 46 | 7 | 14 | 3 | 4 | 2 | 18 |

DFR, Disease free after local recurrence; ADM, alive with metastases; DOD, died of disease.

months). Four of them (40%) could be cured by renewed resection; three after CR and one after IH.

Local recurrence was found in two patients who underwent surgical procedure with a wide margin (7.4%), whereas in six after resection with a marginal margin. Thus, the incidence of local recurrence was 37.5%, when a marginal margin was achieved. Local recurrence rate was not influenced by the tumor stage and not significantly by the tumor volume (22.% in large tumors versus 20% in small tumors).

Distant metastases were found in 17 cases (35.4%), mostly recorded as pulmonary metastases ($n$=15). The mean interval was 17.2 months (range 3–48 months). Sixteen distant metastases occurred in stage IIB tumors, whereas only one occurred in a stage IB tumor. The incidence of distant metastases was not influenced by the surgical margin achieved. Distant metastases occurred more frequently in large tumors (38.7%) than in small tumors (33.3%), but the difference was not significant.

The incidence of local recurrence was dependent significantly upon the achieved surgical margin, whereas the incidence of distant metastases correlates with the tumor stage ($p$=0.05).

*Survival analysis*

The overall survival analysis in relation to the tumor stage and the surgical margin achieved are demonstrated in Figures 4 and 5.

The best prognosis is seen in low-grade chondrosarcomas with a 5-year survival of 95%, whereas high-grade tumors survive only in 21%. The difference was statistically significant ($p$=0.05).

The difference of 5-year survival between achieved wide margin (77%) and marginal margin (62.5%) was not significant. However, comparing intralesional resection (0% 5-year survival) with marginal or wide resection shows a significant difference ($p$=0.05).

Survival was not influenced by the tumor volume, the origin of the tumor, or the performed surgical procedure.

*Complications*

There were two general (3.9%) and 20 local complications (39.2%). Two patients died peri-operatively; one due to pulmonary embolism 10 days after surgery, and one due to multiple organ failure 1 month post-operatively.

Local infections and wound healing problems were seen in 10 cases (18%); four after H (23%), three after Ih (17.6%) and three after CR (13%).

After IH, four dislocations of the hip joint (23.5%) could be treated by closed reduction. Four cases of implant loosening, two after CR and two after IH, required removal of the implant in two cases; re-fixation was performed in one case after IH and in one case after CR.

In one patient, peri-artiuclar ossification around the hip joint after IH was resected, but range of motion of the hip joint remained restricted. Femoral nerve palsy occurred in one patient after CR.

The complication rate was not significantly dependent upon the different surgical treatment groups (i.e. CR, IH, and H).

*Functional outcome*

The functional results of the 28 survivors obtained at the last follow-up are shown in Table 6 in relation to the performed surgical procedure. The average follow-up of these patients was 110 months (range,

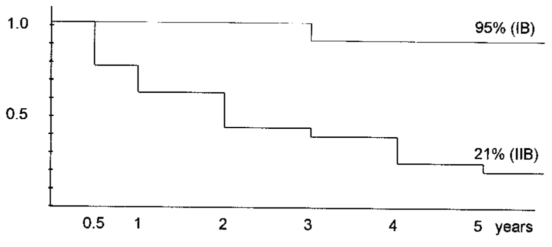

**Fig. 4.** *Kaplan–Meier survival analysis in relation to tumor stage (stage III tumors were excluded).*

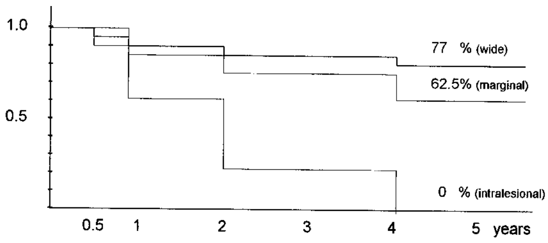

**Fig. 5.** *Kaplan–Meier survival analysis in relation to surgical margin (stage III tumors were excluded).*

4–229 months). Good and excellent results were seen in 19 cases (67.8%), fair in three (10.7%), and poor in six (21.4%).

The mean MSTS score was 69.2% (range, 26.6–100%). The best functional results were seen after CR with a mean score of 79.5% (range, 40–100%). The score was not significantly reduced in cases of IH (61.4%; range, 26.6–100%). As expected, all patients who underwent hemipelvectomy showed poor functional results with a mean score of 37.6% (range, 33–40%). The different of MSTS scores between H and CR or IH were statistically significant ($p$=0.05).

### Discussion

Chondrosarcoma arises in the pelvis in approximately 40–50% of all chondrosarcomas.[1,2,4–11,13–16]

Because there are no anatomical barriers in the pelvis against expansion of the tumor, most pelvic sarcomas produce a large extraskeletal mass and have to be classified as extracompartmental.

A few articles[6,7,13,15] have analyzed the prognostic factors that determine the outcome of patients with chondrosarcoma of the pelvis. In our series, the surgical margin obtained and the tumor stage were the only two significant prognostic factors. Like reports by other authors,[6,7,13] the tumor volume had no influence. In contrast to previous reports,[7] the tumor's epicenter was also not a prognostic factor in our series.

An adequate surgical margin (i.e. wide or radical margin) is considered the most important factor associated with local tumor control.[1–13,15] In the pelvis, a wide margin is often difficult to obtain due to adjacent visceral, vascular or neural structures. The type of surgical treatment (limb sparing procedure versus hemipelvectomy) had no influence on the achieved surgical margin.[5–7,13,15] It is the adequacy of the surgical margin and not the type of surgical procedure that is critical for local tumor control.

The role of tumor grade for local tumor control in chondrosarcomas is not clear. There exists only one report[7] about the prognostic factor of tumor grade upon local failure in chondrosarcoma of the pelvis. Our findings have confirmed most previous studies[8–11,13,15] that could not find a relation between incidence of local recurrence and tumor grade.

However, the overwhelming influence of tumor

**Table 6**. *Functional results in 28 survivors of pelvic chondrosarcoma*

| Surgical procedure | *n* | Overall Enneking score[20] Excellent | Good | Fair | Poor | Mean MSTS score[20] (range) |
|---|---|---|---|---|---|---|
| Continuity resection | 16 | 10 | 2 | 3 | 1 | 79.5% (40–100%) |
| Internal hemipelvect. | 9 | 2 | 5 | | 2 | 61.4% (26.6–100%) |
| Hemipelvectomy | 3 | | | | 3 | 37.6% (33–40%) |
| Total | 28 | 12 | 7 | 3 | 6 | 69.2% (26.6–100%) |

MSTS, Musculoskeletal Tumor Society.

stage on systemic failure (i.e. incidence of distant metastases) indicates its significance as a prognostic factor that determines survival. In this regard, our results agree with all previously published reports chondrosarcoma at all sites[1–5,8,10–12] and in the pelvis.[6,7,9,13–15]

Pritchard *et al.*[12] reported a better survival in patients who underwent limb sparing procedures than in those who underwent hemipelvectomy. They attributed this to a subtle selection bias, because the only differences between the two groups were in the mean age and sex. However, more patients with large high-grade tumors underwent hemipelvectomy. On multivariate analysis, tumor stage was the only significant prognostic factor.

The type of surgical procedure that should be performed in pelvic malignancies depends mainly upon the tumor localization and extension. For local tumor control, the primary goal of resection is to achieve an adequate surgical margin.[2,5,12,17] One of the most critical areas to obtain an adequate margin is the sacroiliac region. The findings of a positive surgical margin in the residual sacrum are consistent with previous observations about pelvic malignancies.[4–7,9,13,15] Further studies using magnetic resonance imaging and CT scan have to be focused on this area in question to stage, pre-operatively, the exact sacral involvement of the tumor. The sectioning of sacral roots or even a total sacrectomy should be considered to control the tumor. These more radical resections are easier in conjunction with extended hemipelvectomy. Hemipelvectomy is further indicated, when the sciatic nerve has to be sacrified, the tumor invades the iliac vessels, or when resection of large amounts of muscles leave the leg with poor function.[7,9,13,15]

When the tumor involves the ileum or the anterior pelvic girdle without acetabular involvement, continuity resection[4,5] is indicated. Restoration of the pelvic girdle using allografts, autografts or composite fixations is necessary when the continuity of the dorsal pelvic girdle is interrupted.

In acetabular lesions, internal hemipelvectomy and endoprosthetic replacement by computer-aided design prosthesis,[2,9,13,14,21] polyacetal pelvic replacement[15] or by the saddle prosthesis[22] ensure a primary fixation with good functional results.

If, however, major parts of the ilium have to be resected and proximal anchorage of the prosthesis cannot be achieved, long-term stability cannot be guaranteed. When proximal parts of the ilium can be secured, new prosthetic devices with an intramedullary fixation by a spongy shaft may provide good function and secondary fixation by bone ingrowth.[21]

Besides the tumor localization, other factors like the functional result, the complication rate and the oncologic prognosis judged the tumor stage may influence the choice of the surgical procedure.

Local complications after resection of pelvic malignancies are common and have to be expected in about 50% of cases.[4,5,7,13,14] In our series, the local complication rate was 39.2%. Most local complications were caused by wound healing problems like skin necrosis, sinuses and hematomas. In our series, they were not significantly influenced by the surgical procedure.

The reasons for the high complication rate are not influenced by the type of reconstruction, but rather by the extent of the necessary surgical resection. Local complications are considered to be attributed to the large cavities due to extensive approaches and the frequent tumor involvement of gluteal muscles or the sciatic notch requiring transection of the glutal or internal iliac vessels. Because of the often extensive blood loss resulting in impaired coagulation, a routinely performed second look operation washing out the hematomas should be recommended in the postoperative management after resection of pelvic malignancies, and may help to reduce the local complication rate.

The best functional results in our series were achieved following continuity resections with a mean MSTS score of 79.5% followed by internal hemipelvectomy and endoprosthetic replacement in acetabular lesions with a mean score of 61.4%. These findings are comparable with previous reports.[4,5,9,11,13–15]

More restricted alternative procedures like iliofemoral arthrodesis or pseudarthrosis[24] result in either a considerable shortening of the leg or limited mobility. Reconstruction by the saddle prosthesis[22] may be a good compromise between the achievable function and the extent of the surgical procedure.

In low-grade pelvic chondrosarcomas, the excellent oncologic prognosis (5-year survival of 95%) justifies adequate surgery achieving wide margins and restoration of the pelvic girdle by continuity resection or internal hemipelvectomy and endoprosthetic replacement. In high-grade chondrosarcomas of the pelvis, systemic failure led to a shortened survival in the majority of patients (mean 5-year survival of 21% in our series).

Because of this poor prognosis, however, the question 'how much' surgery is justified and necessary in high-grade chondrosarcomas is still not answered and extensively discussed.[4,5,7,15,22–24]

Because of the high incidence of metastases in high-grade chondrosarcomas, there remains the hope for an effective systemic therapy.[7]

### References

1 Gitelis S, Bertoni BF, Picci, Campanacci M. Chondrosarcoma of bone. The experience at the Instituto Orthopedico Rizzoli. *J Bone J Surg* 1981; 63A:1248–57.
2 Healey JH, Lane JM. Chondrosarcoma. *Clin Orthop* 1986; 205:119–29.
3 Springfield DS, Gebhardt MC, McGuire MH. Chondrosarcoma: a Review. *J Bone J Surg* 1996; 78A:141–9.
4 Campanacci M, Capanna R. Pelvic resections: the Rizzoli Institute experience. *Orthop Clin North Am* 1991; 22:45–86.
5 Enneking WF, Dunham WK. Resection and reconstruction for primary neoplasms involving the innominate bone. *J Bone J Surg* 1978; 66A:731–46.
6 Ozaki T, Hillmann A, Lindner N, Blasius S, Winkelmann W. Chondrosarcoma of the pelvis. *Clin Orthop* 1997; 337:226–39.
7 Sheth DS, Yasko AW, Johnson ME, Ayala AG, Murray JA, Romsdahl MM. Chondrosarcoma of the pelvis. Prognostic factors for 67 patients treated with definitive surgery. *Cancer* 1996; 78:745–50.
8 Evenas HL, Ayala AG, Romsdahl MM. Prognostic factors in chondrosarcoma of bone. A clinicopathologic analysis with emphasis on histologic grading. *Cancer* 1977; 40:818–31.
9 Kawai A, Healey JH, Boland PJ, Lin PP, Huvos AG, Meyers PA. Prognostic factors for patients with sarcomas of the pelvic bones. *Cancer* 1998; 82:851–9.
10 Kreicbergs A, Boquist L, Borssen B, Larsson SE. Prognostic factors in chondrosarcoma. *Cancer* 1982; 50:577–83.
11 van Loon CJM, Veth RPH, Pruszczynski M, Wobbes T, Lemmens JAM, van Horn J. Chondrosarcoma of bone: oncologic and functional results. *J Surg Oncol* 1994; 57:314–21.
12 Pritchard DJ, Lunke RJ, Tylor WF, Dahlin DC, Medley BE. Chondrosarcoma: a clinocpathologic and statistical analysis. *Cancer* 1980; 45:149–57.
13 Shin KO, Rougraff BT, Simon MA. Oncologic outcome of primary bone sarcoma of the pelvis. *Clin Orthop* 1994; 304:307–17.
14 Windhager R, Karner J, Kutschera HP, Polterauer P, Salzer-Kuntschik M, Motz R. Limb salvage in periacetabular sarcomas. Review of 21 consecutive cases. *Clin Orthop* 1996; 331:265–76.
15 Marcove RC, Mike V, Hutter RVP. Chondrosarcoma of the pelvis and upper end of the femur: an analysis of factors influencing survival time in 113 cases. *J Bone J Surg* 1972; 54A:561–72.
16 Mutschler W, Burri C, Kiefer F. Functional evaluation after pelvic resections with endoprosthetic replacement. In: Enneking WF, ed. *Limb Salvage in Musculoskeletal Oncology*. New York: Churchill-Livingstone, 1987:156–66.
17 Enneking WF, Spanier SS, Goddman MA. A system for surgical staging of musculoskeletal sarcoma. *Clin Orthop* 1980; 153:106–20.
18 Göbel V, Jürgens H, Etspüler G *et al.* Prognostic significance of tumor volume in localized Ewing's sarcoma of bone in children and adolescents. *J Cancer Res Clin Oncol* 1987; 113:187–91.
19 Kaplan EL, Meier P. Nonparametric estimation for incomplete observations. *J Am Stat Assoc* 1958; 53:457–81.
20 Enneking WF, Dunham W, Gebhardt MC, Malawer M. Pritchard DJ. A system for the functional evaluation of reconstructive procedures after surgical treatment of tumors of the musculoskeletal system. *Clin Orthop* 1993; 286:241–6.
21 Gradinger R, Rechl H, Ascherl R, Plötz W, Hipp E. Endoprosthetic replacement of the pelvis in malignant bone tumors. *Orthopäde* 1993; 22:167–73.
22 Aboulafia AJ, Buch R, Mathews J, Li W, Malawer MM. Reconstruction using the saddle prosthesis following excision of primary and metastatic periacetabular tumors. *Clin Orthop* 1995; 314:203–13.
23 Abudu A, Grimer RJ, Cannon SR, Carter SR, Sneath RS. Reconstruction of the hemipelvis after excision of malignant tumors. *J Bone J Surg* 1997; 79B:773–9.
24 O'Connor MI, Sim FH. Resection of pelvic sarcomas and reconstruction with nonprosthethic techniques. *Orthopäde* 1993; 22:174–8.
